# Optimal reimplantation timing in two-stage exchange for periprosthetic joint infection: an observative cohort study in Asian population

**DOI:** 10.1186/s12891-023-07129-8

**Published:** 2024-01-02

**Authors:** Meng-Lun Tsai, Allen Herng-Shouh Hsu, Cheng-Ta Wu, Po-Chun Lin, Timothy L Tan, Feng-Chih Kuo

**Affiliations:** 1 Department of Orthopaedic Surgery, Kaohsiung Chang Gung Memorial Hospital, No. 123, Dapi Road, Niaosong District, 833 Kaohsiung, Taiwan; 2 Department of Orthopaedic Surgery, University of California, San Francisco, San Francisco, CA USA; 3 College of Medicine, Chang Gung University, Kaohsiung, Taiwan; 4 Center for General Education, Cheng Shiu University, Kaohsiung, Taiwan

**Keywords:** Reimplantation, Periprosthetic joint Infection, Timing of reimplantation, Time to reimplant, Asian, Exchange arthroplasty, 2-stage arthroplasty

## Abstract

**Background:**

The optimal timing for reimplantation for periprosthetic joint infection (PJI) has not been established and varies from a few weeks to several months. The aim of this study was to assess the commendable time between implant removal and reimplantation in patients who underwent two-stage exchange arthroplasty for PJI.

**Methods:**

We retrospectively reviewed 361 patients who were treated with two-stage exchange arthroplasty for hip and knee chronic PJI at our institution between January 2000 and December 2018. Patient characteristics, comorbidities, surgical variables, microbiology data, and time to reimplantation were recorded. All patients were followed for a minimum of one year. Treatment failure was defined by Delphi criteria. Logistic regression analyses were used to calculate survival rates and adjusted odds ratios (ORs) of treatment failure.

**Results:**

In final analysis, 27 (7.5%) had treatment failure. Factors related to treatment failure including interim spacer exchange (OR, 3.13; confidence interval (CI), 1.04–9.09, *p* = 0.036), higher ESR level at reimplantation (OR, 1.85; CI, 1.05–3.57; *p* = 0.04), and time to reimplantation (OR, 1.00; CI, 1.003–1.005, *p* = 0.04). Performing revision arthroplasty surgery from 16 to 20 weeks had highest successful rate. The reimplantation over 24 weeks had a lower successful rate. However, no statistical significance in comparing each interval group.

**Conclusion:**

Our study emphasized the importance of timely reimplantation in achieving successful outcomes. Factors such as ESR levels, spacer exchange, and the duration of time to reimplantation influenced the likelihood of treatment failure in two-stage exchange arthroplasty for hip and knee PJI.

## Introduction

Periprosthetic joint infection (PJI) of the hip and the knee is one of the catastrophic complications of primary total joint arthroplasty (TJA) [1]. However, the management of PJI remains in debate and surgeons constantly modify surgical protocols and optimize host risk factors to improve treatment outcomes [2–8].

The favorable surgical treatment for PJI has trended toward 2-stage exchange arthroplasty in North America [9, 10]. The 2-stage exchange protocol generally consists of a first stage, during which the infected prosthesis is explanted, complete debridement and a microorganism-tailored antibiotic-loaded cement spacer is placed into the joint [11–13]. After that, a second stage is to reimplant a new prosthesis. However, absolute eradication of a periprosthetic infection is difficult to achieve owing to sensitivity and specificity of current pre-operative diagnostic tests cannot reach 100%. Therefore, the optimal timing for reimplantation remains controversial and not been established according to 2018 International Consensus Meeting (ICM) on PJI [14]. Previous study discovered that the length of the interstage interval is not a statistically significant predictor of failure in patients undergoing 2-stage arthroplasty for PJI [15], whereas interval between spacer insertion and reimplantation have been widely discussed [16, 17]. The optimal timing for the reimplantation has not been established within the recent literature and varies from a few weeks to several months or even years. The optimal timing of reimplantation also varies between counties.

The aim of this study was to assess the commendable time between implant removal and reimplantation in patients who underwent a two-stage joint arthroplasty for PJI.

## Materials and methods

This Institutional Review Board (IRB) of Chang Gung Memorial Hospital, Taiwan approved the study and waived the requirement to obtain informed consent (Protocol no. 202200792B0, Date: 2022/05/30). This study was conducted in line with the principles of the Declaration of Helsinki. we retrospectively reviewed 361 patients who were treated with two-stage exchange arthroplasty for hip and knee chronic PJI at our institution between January 2000 and December 2018. Patients without undergoing subsequent reimplantation (242 cases) and those with native joint infection treated with two-stage exchange arthroplasty (79 cases) were excluded. Patients who were expired not owing to PJI (51 case), followed up < 1 year (42 cases) and those without 2-weeks antibiotics holidays (9 cases), were excluded. Eventually, a total of 361 patients (174 knees and 187 hips) were include in the final cohort.

**Fig. 1:**
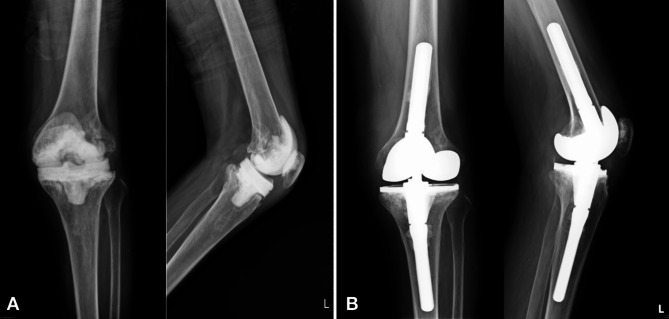
Two-Stage Exchange Arthroplasty in a 66-Year-Old Female Patient with Left Knee Periprosthetic Joint Infection. The patient underwent a two-stage exchange arthroplasty, and the reimplantation was performed after 149 days. Following a two-year follow-up, the patient remained free from infection. **A**: The first stage of resection arthroplasty, showing an antibiotic-loaded bone cement spacer in place. **B**: The second stage of reimplantation with a cemented prosthesis

**Fig. 2:**
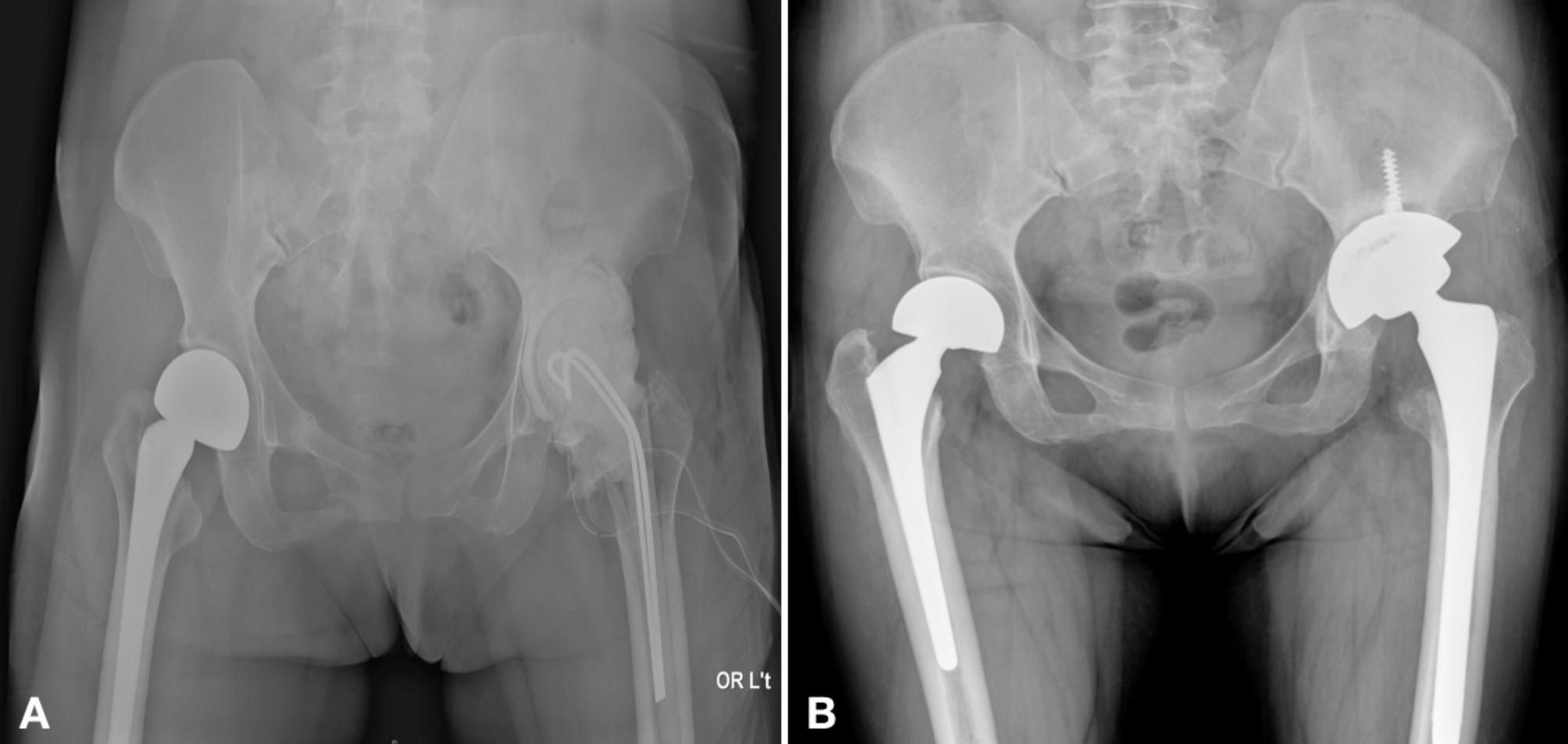
Two-Stage Exchange Arthroplasty in a 47-Year-Old Female Patient with Left Hip Periprosthetic Joint Infection. This patient underwent a two-stage exchange arthroplasty with a 58-day interval to reimplantation. She remained free from infection during a three-year follow-up period. **A**: The first stage of resection arthroplasty featuring an antibiotic-loaded bone cement spacer. **B**: The second stage of reimplantation using a cementless prosthesis

The PJI was diagnosed according to the definition of 2013 Musculoskeletal Infection Society (MSIS) criteria [18]. During the first stage, radical debridement, removal of all prosthesis, and implantation of antibiotic-loaded cement beads or spacer were performed. The regimen of antibiotics in the bone cement was determined according to the culture results from preoperative joint aspiration or previous culture report. If the infecting microorganism could not be confirmed at the time of resection arthroplasty, empirical combination of 2–4 g vancomycin and 2–4 g piperacillin or ceftazidime were applied per 40-g bone cement. If the infection could not be controlled during the interim stage, further debridement and spacer exchange were executed. Intravenous organism-specific antibiotics were given postoperatively at least 2–4 weeks, followed by oral antibiotics for two weeks [19–22]. All reimplantation were performed after an at least 2-week antibiotic holiday. The timing of reimplantation was on the basis of resolution of symptoms, no elevation of erythrocyte sedimentation rate (ESR) and serum C-reactive protein (CRP), and radiographic findings suggesting no ongoing infection [23]. In patients with underlying diseases such as gout, rheumatic arthritis, or other chronic diseases, ESR and serum CRP level may not show normal level even if infection was under control. In these cases, the indication of reimplantation was according to the clinical condition and a decreasing trend of ESR and CRP. During the second stage, new prostheses were reimplanted and cemented with antibiotic-loaded bone cement for knee implantation (Fig. 1). For hip reimplantation, the cementless or cemented prosthesis were chosen by the surgeon’s intraoperative decision (Fig. 2). All operation were executed by high-volume arthroplasty surgeons in a single institution.

Details of characteristics of interest including age, gender, body mass index (BMI), joint (hip or knee), laterality (left or right), surgery (primary or revision), American Society of Anesthesiologists (ASA) classification, polymicrobial, resistant organism, CRP and ESR level at the reimplantation, interim spacer exchange and time to reimplantation were extracted. The treatment failures at 1 year were defined according to the Delphi consensus [24].

All data were analyzed using R project version 4.0.5. Continuous variables were presented with mean and standard deviation (SD) and median with minimum and maximum. Categorical variables are described as number and percentages. To identified whether there was a most appropriate cut point for reimplantation, Fisher’s exact test or chi-square test was used for evaluating treatment failures and successes of foundational characteristics. Logistic regression was conducted to research the optimal interval of time to reimplantation, and variables which p value less than 0.2 were included in logistic regression analysis. Post hoc power analysis for logistic regression was performed and the alpha error probability was set as 0.05. After evaluation based on current information, one minus the probability of the beta error was = 0.90, and which meant that the sample size was adequately powered at 90%. P value less than 0.05 indicates statistically significant.

## Results

After 1-year follow-up, 334 patients (92.5%) had treatment success and 27 (7.5%) had treatment failure based on the Delphi criteria. Mean age was 62.6 (SD ± 12.5) and mean BMI was 26.8 (SD ± 5.16). One hundred and seventy-four patients underwent total knee reimplantation and 158 patients (90.8%) had treatment success. For hip reimplantation, 187 patients complete second stage reimplantation with a 94.1% of treatment success. Mean ESR level at reimplantation was 36.0 (SD ± 19.4) mm/hr in the treatment failure group and 24.6 (SD ± 18.6) mm/hr in the treatment success group (*p* = 0.003). Patients with interim spacer exchange had a higher failure rate than those without spacer exchange (70% vs. 29.6%, *p* = 0.005). The average time to reimplantation was 220 (SD ± 271) days in the treatment failure group compared to 143 (SD ± 118) days in the treatment success group (*p* = 0.003). Other predictors did not show statistical difference (Table 1).

**Table 1:** Patient characteristics

	Failure (N = 27)	Success (N = 334)	Total (N = 361)	*p*-value
Age, years				0.098
Mean (SD)	59.1 (12.6)	62.9 (12.5)	62.6 (12.5)	
Median [Min, Max]	59.0 [29.0, 85.0]	65.0 [26.0, 89.0]	65.0 [26.0, 89.0]	
Sex, n (%)				0.286
Female	12 (44.4%)	167 (50.0%)	179 (49.6%)	
Male	15 (55.6%)	167 (50.0%)	182 (50.4%)	
BMI, kg/m^2^				0.210
Mean (SD)	28.7 (6.97)	26.6 (4.96)	26.8 (5.16)	
Median [Min, Max]	27.8 [19.6, 48.6]	26.3 [15.7, 50.0]	26.6 [15.7, 50.0]	
Joint, n (%)				0.016
Hip	11 (40.7%)	176 (52.7%)	187 (51.8%)	
Knee	16 (59.3%)	158 (47.3%)	174 (48.2%)	
Laterality, n (%)				0.430
Left	13 (48.1%)	145 (43.4%)	158 (43.8%)	
Right	14 (51.9%)	189 (56.6%)	203 (56.2%)	
Surgery, n (%)				0.603
Primary	21 (77.8%)	264 (79.0%)	285 (78.9%)	
Revision	6 (22.2%)	70 (21.0%)	76 (21.1%)	
ASA, n (%)				
1	1 (3.7%)	12 (3.6%)	13 (3.6%)	
2	10 (37.0%)	159 (47.6%)	169 (46.8%)	0.492
3	16 (59.3%)	163 (48.8%)	179 (49.6%)	0.311
Polymicrobial, n (%)	3 (11.1%)	27 (8.1%)	30 (8.3%)	0.257
Resistant organism, n (%)	1 (3.7%)	44 (13.2%)	45 (12.5%)	0.129
CRP, mg/L				0.699
Mean (SD)	9.21 (9.18)	6.74 (9.91)	6.92 (9.86)	
Median [Min, Max]	5.60 [0.280, 32.3]	3.49 [0, 69.0]	3.66 [0, 69.0]	
ESR, mm/h				0.003
Mean (SD)	36.0 (26.0)	24.6 (18.6)	25.5 (19.4)	
Median [Min, Max]	28.0 [2.00, 85.0]	21.5 [0, 99.0]	23.0 [0, 99.0]	
Interim spacer exchange, n (%)				0.005
Yes	19 (70.4%)	281 (84.1%)	300 (83.1%)	
No	8 (29.6%)	53 (15.9%)	61 (16.9%)	
Time To Reimplant, days				0.003
Mean (SD)	220 (271)	143 (118)	149 (137)	
Median [Min, Max]	117 [49.0, 1380]	117 [33.0, 1030]	117 [33.0, 1380]	

*Kruskal Wallis Rank Test with α = 0.05 as significant

SD, Standard Deviation; BMI, Body Mass Index; IQR, Interquartile Range; ASA, American Society of Anesthesiology; CRP, C-reactive protein; ESR, erythrocyte sedimentation rate

**Fig. 3:**
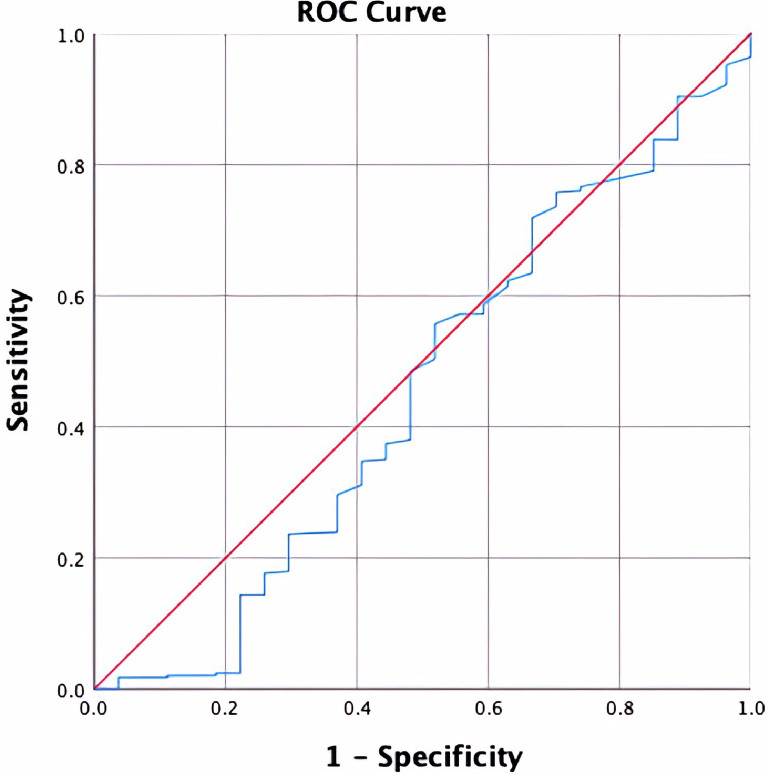
ROC Curve Analysis Evaluating Time to Reimplantation as a Predictor of Failure, with an Area Under the Curve (AUC) of 0.453

The receiver operating characteristic curve (ROC) did not imply a definite threshold time to reimplantation above which patients are at significantly higher risk of failure (area under the curve (AUC) = 0.453; Fig. 3). While in logistic regression, time to reimplantation was a statistically significant predictor of treatment failure (Table 2). We divided all cases into 6 groups (6 to 8 weeks, 8 to 12 weeks, 12 to 16 weeks, 16 to 20 weeks, 20 to 24 weeks, and over 24 weeks). We detected that a dominated higher proportion of cases remained free from reinfection until final follow up in the 16 to 20 weeks groups. However, time to reimplantation in the over 24 weeks groups implied a decreased successful rate (Table 3). However, there is no observed statistical significance when comparing the interval groups individually.

**Table 2:** Association between predictive factors and one-year failure as determined by the Delphi Consensus using logistic regression model

	1-year Delphi failure		
Predictors	Odds Ratios	CI	*p*-value
Joint (Knee)	3.13	1.00–11.11	0.057
Age, year	0.96	0.92–1.00	0.083
Interim spacer exchange	3.13	1.04–9.09	**0.036**
Time to reimplantation, day	1.00	1.001–1.005	**0.040**
Resistant organism	3.95	0.52–29.41	0.186
ESR, mm/hr	1.85	1.05–3.57	**0.040**

CI, confidence interval; BMI, Body Mass Index; CRP, C-reactive protein; ESR, erythrocyte sedimentation rate

**Table 3:** Comparation between each time to reimplantation group

		1-year Delphi success			
Time to reimplantation	N	Success rate (%)	Odds Ratios	95% CI	*p-*value
6–8 weeks	16	93.8	Reference		
8–12 weeks	64	90.6	0.64	0.07–5.77	0.694
12–16 weeks	87	93.1	0.90	0.10–8.02	0.925
16–20 weeks	80	95	1.27	0.13–12.14	0.838
20–24 weeks	31	93.5	0.97	0.08–11.54	0.979
Over 24 weeks	78	89.7	0.58	0.07–5.02	0.624

*p-value less than 0.05 as significant

N, case number; CI, confidence interval

### Complication

**Fig. 4:**
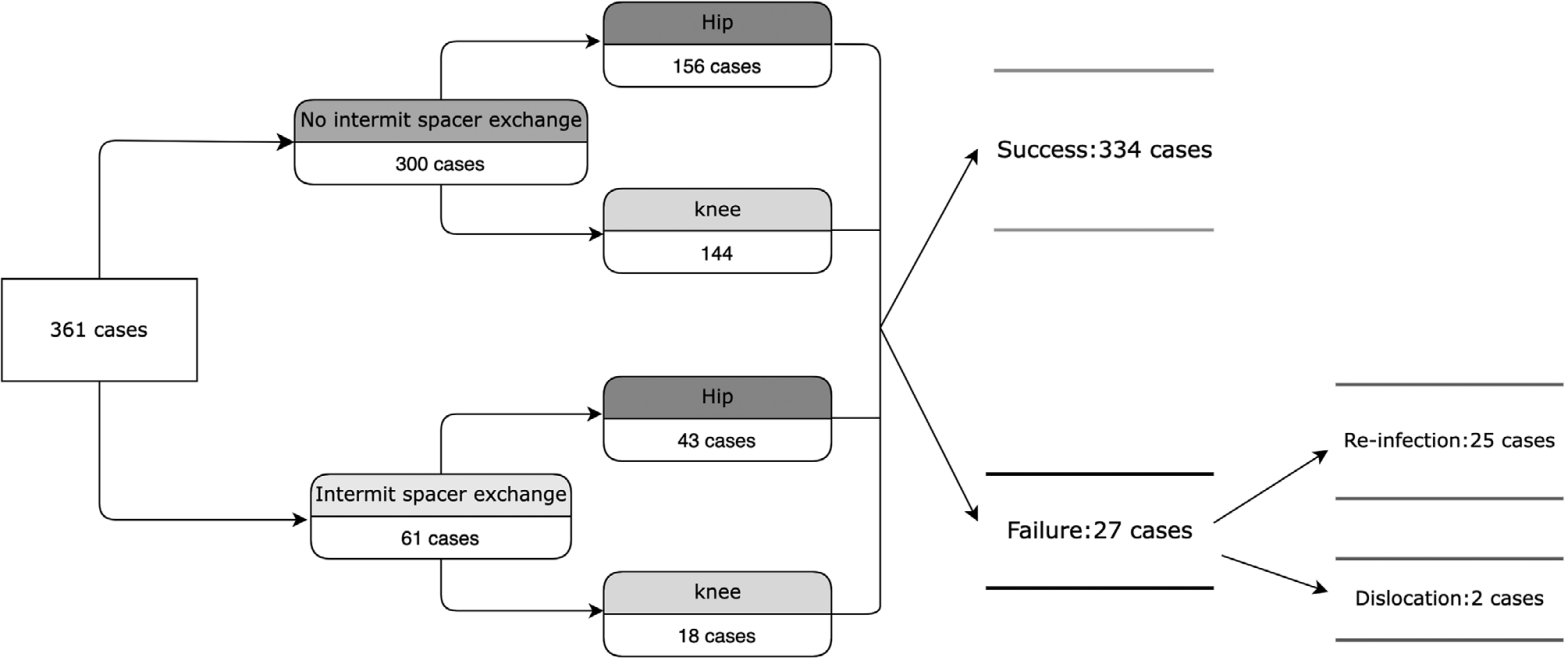
Overview of Complications Encountered in This Study

All post-operative complications were documented in this study (Fig. 4). Positive microbiology results were noted in 13 revision total knee arthroplasty (TKA) and 10 revision total hip arthroplasty (THA) patients during the follow-up period. Two cases of reinfection revealed culture negative microbiology results. Three patients had recurrent infection after repeated two-stage exchange arthroplasty and all these patients were treated with permanent resection arthroplasty. Two patients had encountered recurrent dislocation and revision total joint arthroplasty were performed.

## Discussion

Two-stage exchange arthroplasty is the well-known and preferred surgery for PJI, which remains a challenging topic due to miscellaneous clinical presentations and manifestations, and lack of randomized controlled trials [9]. One of the debating issues of two-stage exchange surgery is concerned with the appropriate timing of reimplantation. Timing to reimplantation is still inconclusive from weeks to a few months [25, 26]. To our knowledge, the number of our cases was relatively large in current literature (Table 4). We believed that our study may provide the satisfactory recommendation for the optimal timing of revision arthroplasty. In our study, we suggested that the timing of highest successful rate for reimplantation was 16 to 20 weeks after the first stage surgery. The interval more than 24 weeks had a lowest successful rate compared to other interval time. However, there was no statistical significance in comparing each interval group, which implied no clear association between specific time to reimplantation and treatment success following 2-stage exchange arthroplasty.

**Table 4:** Comparison of current reported literatures on time to reimplantation

Study	Country	Year	No. of patients	Failure (%)	Outcome
McDonald et al. [26]	America	1989	82	11 (13.4%)	Over 1 year: 7.1% (4 of 56 patients) Less than 1 year: 26.9% (7 of 26 patients)
Lieberman et al. [27]	America	1994	32	3 (9%)	No difference between the 6 weeks protocol and over one year protocol.
Haddad et al. [27]	United Kingdom	2000	50	4 (8%)	No increased rate of failure in most case of at least 3-weeks inter-period.
Sabry et al. [29]	America	2014	314	105 (33.4%)	Increased interstage interval was associated with a higher infection rate. Failure:105 cases (average 124 days) / Success: 209 cases (average 96 days)
Ines et al. [30]	Austria	2015	76	19 (30.3%)	The optimal timing for second-stage surgery was between 4 and 11 weeks.
Arash et al. [15]	America	2018	282	63 (22.3%)	Length of interstage duration was not a statistically significant predictor. Interstage over 26 weeks had higher failure rate.
Tobias et al. [31]	Germany	2019	38	1 (2.6%)	Short interval (average 18 days) had a similar outcome with long interval (average 63 days)
This study	Taiwan	2023	361	27 (7.5%)	The timing of highest successful rate for second-stage surgery was between 16 and 20weeks. Internal over 24weeks groups implied decreased successful rate. No statistical difference between each group.

Prior studies have a conflict result about the optimal timing for reimplantation (Table 4). McDonald et al. [26] found that infection rate of reimplantation performed over 1 year after resection arthroplasty was 7.1% (4 of 56 patients), compared with 26.9% (7 of 26 patients) in the procedure performed less than 1 year. On the contrary, Lieberman et al. found no difference in failure rates for patients who were reimplanted in the 6-week interstage protocol with those who were in the over 1-year protocol [27]. Haddad et al. also reported no increased rate of failure in most case of at least 3-weeks inter-period to implantation [28]. In other respects, Sabry et al. reported an increased interstage interval between resection and reimplantation was associated with higher recurrent infection rates in a cohort study (314 infected TKAs) [29]. Their median interval between stages was 103 days (range, 2-470 days). Arash et al. found that the length of interstage duration was not a statistically significant predictor of failure in 2-stage exchange arthroplasty for PJI (282 patients) [15]. The average time to reimplantation of above study was 100.2 days (range, 20–648), and the data demonstrated that patients reimplanted at > 26 weeks were almost twice as likely to fail compared with those reimplanted in < 26 weeks. Ines et al. reported that the optimal timing for second-stage surgery of PJI is between 4 and 11 weeks (76 cases), 90% of cases were without infection until final follow-up (20.5 (range 0 to 78 months) [30]. The study of 38 patients from Tobias et al. suggests that two-stage revision arthroplasty with short interval (a mean interval of 17.9 days) has a similar outcome than with long interval (a mean interval of 63.0 days) [31]. Compared to the above studies, our study discovered that the reimplantation time in 16 to 20 weeks had highest successful rate compared to another interval group. Our average time to reimplantation is 149 days. Furthermore, the length of interstage of interval over 24 weeks showed increasing failure rate of reimplantation. Nevertheless, no definite statistical evidence indicated the specific timing to perform reimplantation.

There are several limitations of our studies. First, this is a retrospective study depending on existing data, which may have introduced bias. Moreover, we lacked long term follow up information. Third, we were unable to evaluate certain variables, such as functional score, malnutrition, and associated comorbidities. Over two well experienced surgeons performed exchange procedures, which may also have interindividual discrepancies in surgical techniques, that may generate undetectable bias in our study.

## Conclusion

In summary, the statistical findings emphasized the importance of timely reimplantation in achieving successful outcomes. Factors such as ESR levels, spacer exchange, and the duration of time to reimplantation emerged as critical variables influencing the likelihood of treatment failure in two-stage exchange arthroplasty for hip and knee PJI. However, the precise timing for reimplantation remains uncertain, and further research is necessary to reach an indisputable conclusion.

## Data Availability

The datasets used and/or analyzed during the current study are available from the corresponding author and first author on reasonable request.

## Abbreviations

PJI: Periprosthetic Joint Infection
ORs: Odds Ratios
TJA: Total Joint Arthroplasty
TKA: Total Knee Arthroplasty
THA: Total Hip Arthroplasty
ICM: International Consensus Meeting
MSIS: Musculoskeletal Infection Society
ESR: Erythrocyte Sedimentation Rate
CRP: C-reactive Protein
BMI: Body Mass Index
ASA: American Society of Anesthesiologists
SD: Standard Deviation
AUC: Area Under the Curve
ROC: Receiver Operating Characteristic
